# Intranasal oxytocin suppresses seizure-like behaviors in a mouse model of NGLY1 deficiency

**DOI:** 10.1038/s42003-024-06131-7

**Published:** 2024-04-22

**Authors:** Yukimasa Makita, Makoto Asahina, Reiko Fujinawa, Hiroshi Yukitake, Tadashi Suzuki

**Affiliations:** 1 Takeda-CiRA Joint Program, 26-1, Muraoka-Higashi 2-chome, Fujisawa, Kanagawa 251-8555 Japan; 2 Global Advanced Platform, R&D Research, Takeda Pharmaceutical Co., Ltd. 26-1, Muraoka-Higashi 2-chome, Fujisawa, Kanagawa 251-8555 Japan; 3 Glycometabolic Biochemistry Laboratory, Cluster for Pioneering Research, RIKEN, 2-1 Hirosawa, Wako Saitama, 351-0198 Japan

**Keywords:** Epilepsy, Molecular neuroscience

## Abstract

NGLY1 deficiency is a genetic disease caused by biallelic mutations of the *Ngly1* gene. Although epileptic seizure is one of the most severe symptoms in patients with NGLY1 deficiency, preclinical studies have not been conducted due to the lack of animal models for epileptic seizures in NGLY1 deficiency. Here, we observed the behaviors of male and female *Ngly1*^−/−^ mice by video monitoring and found that these mice exhibit spontaneous seizure-like behaviors. Gene expression analyses and enzyme immunoassay revealed significant decreases in oxytocin, a well-known neuropeptide, in the hypothalamus of *Ngly1*^−/−^ mice. Seizure-like behaviors in *Ngly1*^−/−^ mice were transiently suppressed by a single intranasal administration of oxytocin. These findings suggest the therapeutic potential of oxytocin for epileptic seizure in patients with NGLY1 deficiency and contribute to the clarification of the disease mechanism.

## Abstract (web summary)

Epileptic seizure is one of the symptoms in patients with NGLY1 deficiency, a congenital disorder of deglycosylation. Here the authors show suppressive effects of oxytocin on seizure-like behaviors in a mouse model of NGLY1 deficiency.

## Introduction

Peptide:*N*-glycanase (NGLY1) is an enzyme that removes the *N*-linked glycans from misfolded *N*-glycoproteins^1,2^. NGLY1 is mainly localized in the cytoplasmic side of the endoplasmic reticulum (ER) and involved in the ER-associated degradation of misfolded *N*-glycoproteins, which plays a crucial role in the quality control of newly synthesized *N*-glycoproteins^3,4^. NGLY1 also activates the transcription factor NRF1 by deglycosylating its glycan chains and substituting their amino acid from asparagine to aspartic acid residues^5,6^.

NGLY1 deficiency is a genetic disease caused by biallelic mutations in the *Ngly1* gene^7^. Patients with NGLY1 deficiency exhibit several symptoms, including developmental delay, intellectual disability, movement disorders, alacrima, and epileptic seizures^8^. The first patient with NGLY1 deficiency was reported in 2012^9^. The number of patients has increased to over 100^10^; however, a satisfactory treatment method has not yet been established for NGLY1 deficiency, probably due to the lack of knowledge on the disease mechanism^11–13^.

We previously established *Ngly1*^−/−^ mice and *Ngly1*^−/−^ rats by deleting the exons 11 and 12 of the *Ngly1* gene. These animals exhibited developmental delay and movement disorders^14,15^. Movement disorders in *Ngly1*^−/−^ rats were significantly improved by a single intracerebroventricular administration of AAV-NGLY1^16,17^. This replacement therapy could be a fundamental treatment, but it requires invasive surgery. Other treatment options are anticipated to reduce patient burden.

For epileptic seizures in patients with NGLY1 deficiency, some approved drugs, including levetiracetam, valproate, lamotrigine, and clobazam, have been tested; however, their efficacies were limited^11^. Researching the disease mechanism is necessary to discover more effective anti-epileptic drugs. The *Ngly1*^−/−^ mouse model is a candidate preclinical model of epileptic seizure in NGLY1 deficiency that is used to uncover the disease mechanism, although it is unknown whether *Ngly1*^−/−^ mice exhibit epileptic seizures.

Herein, we observed the behaviors of *Ngly1*^−/−^ mice by video monitoring and found that these mice exhibited seizure-like behaviors spontaneously. Gene expression analyses and enzyme immunoassay revealed significant decreases in oxytocin in the hypothalamus of *Ngly1*^−/−^ mice. The effects of intranasal oxytocin administration on the seizure-like behaviors in *Ngly1*^−/−^ mice were evaluated to confirm the involvement of oxytocin with seizure-like behaviors in *Ngly1*^−/−^ mice.

## Results

### Spontaneous seizure-like behaviors in *Ngly1*^−/−^ mice

**Fig. 1:**
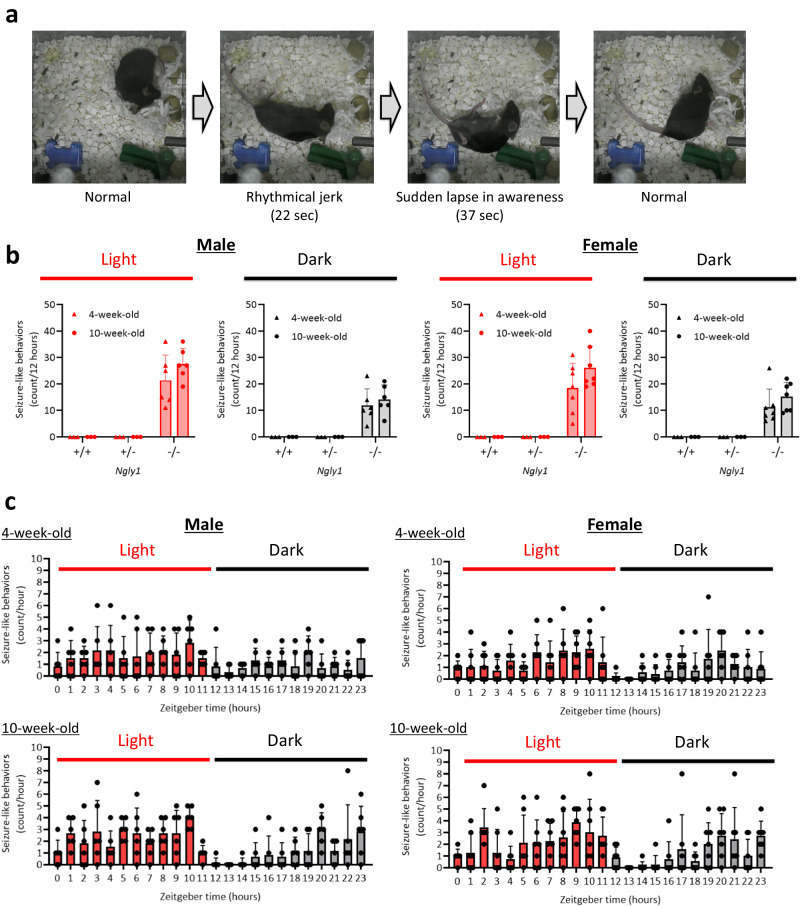
Spontaneous seizure-like behaviors in *Ngly1*^−/−^ mice. **a** Representative photographs of spontaneous seizure-like behaviors in female *Ngly1*^−/−^ mice. **b** Frequencies of seizure-like behaviors in male and female *Ngly1*^+/+^, *Ngly1*^+/−^, and *Ngly1*^−/−^ mice (4 and 10-week-old). Data represents mean and individuals (*N* = 3–7). **c** Hourly variation of seizure-like behaviors in male and female *Ngly1*^−/−^ mice (4 and 10-week-old). Data represent mean + standard deviation and individual values (*N* = 6 or 7).

To clarify whether *Ngly1*^−/−^ mice exhibit epileptic seizures, we observed the behaviors of male and female *Ngly1*^−/−^ mice by video monitoring for 24 h. As shown in the representative figures and video of female *Ngly1*^−/−^ mice, these mice exhibited several seizure-like behaviors spontaneously (Fig. 1a, Supplementary Movie 1). These behaviors seemed to be tonic-clonic or absence seizures, characterized by body stiffness and rhythmical jerking of the limbs or a sudden lapse in awareness. The average frequencies of seizure-like behaviors in 4 and 10-week-old male *Ngly1*^−/−^ mice were 21 and 28 episodes per 12-hour light phase, 12 and 14 episodes per 12-hour dark phase, respectively. Those of female *Ngly1*^−/−^ mice were 18 and 26 episodes per 12 h-light phase, 11 and 15 episodes per 12-hour dark phase, respectively. *Ngly1*^+/+^ and *Ngly1*^+/−^ mice did not exhibit any seizure-like behavior in both light and dark phases at 4 and 10-week-old (Fig. 1b). Although the time zone of occurrence varied individually, almost all mice did not exhibit seizure-like behaviors at the beginning of the dark phase (zeitgeber time 12–13) when they began to move actively (Fig. 1c).

### Transcriptome analyses in the brain and spinal cord of male *Ngly1*^−/−^ mice

**Fig. 2:**
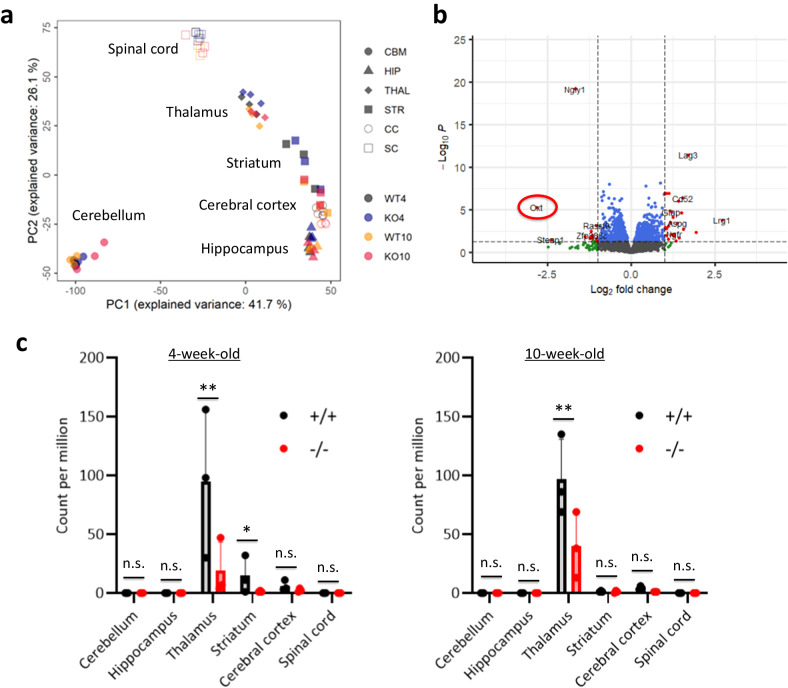
Transcriptome analyses in the brain and spinal cord of *Ngly1*^−/−^ mice. Cerebellum (CBM), hippocampus (HIP), thalamus (THAL), striatum (STR), cerebral cortex (CC), and spinal cord (SC) were collected from male *Ngly1*^+/+^ (WT) and *Ngly1*^−/−^ (KO) mice (4 and 10-week-old). Total RNAs were extracted from each tissue. RNA sequencing was performed on the DNBSEQ platform in Azenta. **a** Principal component analysis plot of all results. *X* and *Y* axes show principal component 1 (PC1) and principal component 2 (PC2) for the data set. The explained variance is indicated in each plot axis. **b** Volcano plot of the results in the thalami of 4-week-old male *Ngly1*^−/−^ mice. *X* axis represents the log2 of the fold change; Y-axis represents the negative decade logarithm of the significance value. Oxytocin (*Oxt*) is marked with a red circle. **c** Expression levels of oxytocin transcripts in 4- and 10-week-old mice. Data represent mean + standard deviation and individual values (*N* = 3). n.s., not significant; *, *p* < 0.05; **, *p* < 0.01 vs. *Ngly1*^+/+^ by Bayes algorithm.

To elucidate the disease mechanism of seizure-like behaviors in *Ngly1*^−/−^ mice, we analyzed the differences in gene expression profiles in the cerebellum, hippocampus, thalamus, striatum, cerebral cortex, and spinal cord of 4 and 10-week-old male *Ngly1*^−/−^ and *Ngly1*^+/+^ mice. Principal component analysis (PCA) of the transcriptome data depicted the differences between these tissues, whereas differences between ages or *Ngly1*^−/−^ and *Ngly1*^+/+^ mice were unclear in the PCA plot (Fig. 2a). However, volcano plots of the transcriptome data revealed significant decreases in oxytocin transcripts in the thalami of 4-week-old *Ngly1*^−/−^ mice (Fig. 2b). Significant decreases in oxytocin transcripts were also detected in the striata of 4-week-old *Ngly1*^−/−^ mice and the thalami of 10-week-old *Ngly1*^−/−^ mice (Fig. 2c). Although 159 Gene Ontology terms were obtained from the enrichment analysis of differentially expressed genes in *Ngly1*^−/−^ mice, NGLY1-related terms, such as proteasome and autophagy, were not included among the 159 terms. Instead, 21 neurotransmission-related terms were included among the 159 terms, suggesting an abnormal neurotransmission in *Ngly1*^−/−^ mice (Supplementary Table 1).

### Specific down-regulation of oxytocin transcripts in the hypothalamus of *Ngly1*^−/−^ mice

**Fig. 3:**
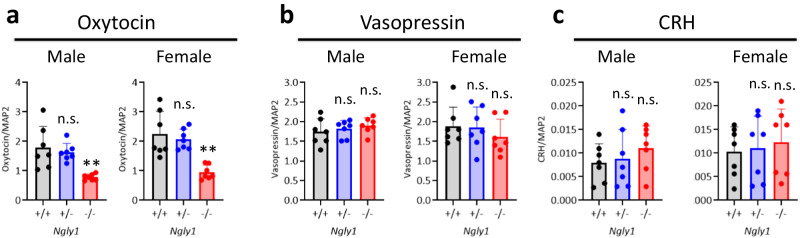
Specific down-regulation of oxytocin transcripts in the hypothalamus of *Ngly1*^−/−^ mice. Hypothalamus was collected from 10-week-old male and female *Ngly1*^+/+^, *Ngly1*^+/−^ and *Ngly1*^−/−^ mice. Transcripts of **a** oxytocin, **b** vasopressin, and **c** CRH were measured using quantitative PCR and normalized to transcripts of the neuronal marker MAP2. Data represent mean + standard deviation and individual values (*N* = 7). n.s., not significant; **p < 0.01 vs. *Ngly1*^+/+^ by Dunnett’s test. CRH, corticotropin-releasing hormone.

The expression levels of oxytocin transcripts were also measured using quantitative PCR (Supplementary Tables 2 and 3) in the hypothalamus of 10-week-old male and female *Ngly1*^−/−^ mice because the hypothalamus is the main production site of oxytocin. The decreased rates of oxytocin transcripts in *Ngly1*^−/−^ mice were 56% (male) and 58% (female) compared to those of *Ngly1*^+/+^ mice. Oxytocin levels were not significantly changed in male and female *Ngly1*^+/−^ mice compared to those of *Ngly1*^+/+^ mice (Fig. 3a). Transcripts of other major hypothalamic neuropeptides, i.e., vasopressin and corticotropin-releasing hormone, were not significantly changed in male and female *Ngly1*^−/−^ mice compared to those of *Ngly1*^+/+^ mice, suggesting that among the hypothalamic neuropeptides, the down-regulation was specific to oxytocin (Fig. 3b, c).

### Oxytocin peptide levels in the hypothalamus, pituitary gland, and plasma of *Ngly1*^−/−^ mice

**Fig. 4:**
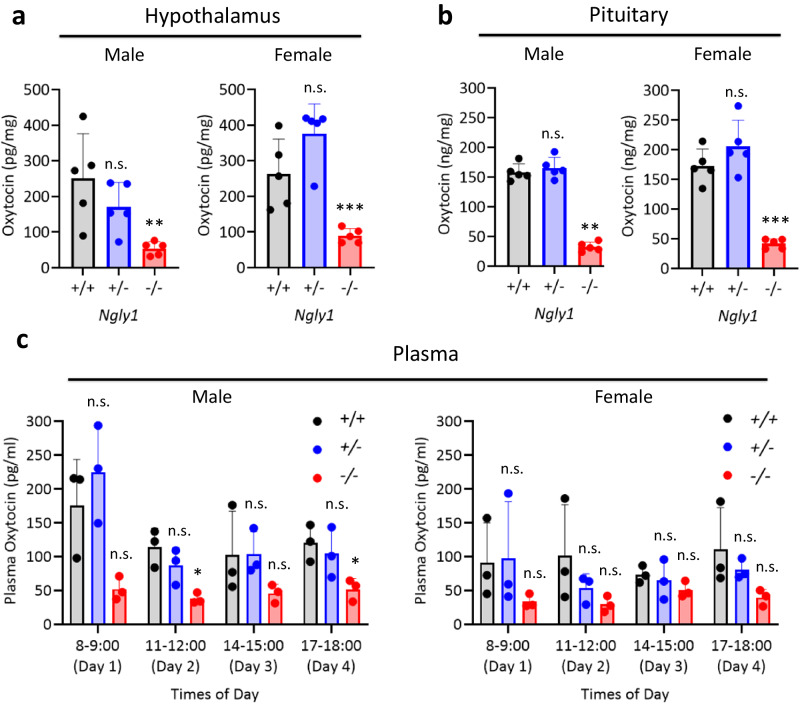
Oxytocin levels in the hypothalamus, pituitary gland, and plasma of *Ngly1*^+/+^, *Ngly1*^+/−^ and *Ngly1*^−/−^ mice. **a** Hypothalamus, and **b** pituitary gland were collected from 10-week-old male or female *Ngly1*^+/+^, *Ngly1*^+/−^, and *Ngly1*^−/−^ mice (*N* = 5). **c** Plasma was collected from 7-week-old male or female *Ngly1*^+/+^, *Ngly1*^+/−^, and *Ngly1*^−/−^ mice (*N* = 3). Oxytocin levels were measured using enzyme immunoassay. Data represent mean + standard deviation and individual values. n.s., not significant; *, *p* < 0.05; **, *p* < 0.01; ***, *p* < 0.001 vs. *Ngly1*^+/+^ by Dunnett’s test.

To confirm oxytocin down-regulation at peptide levels, we quantified oxytocin peptides in the hypothalamus of 10-week-old male and female *Ngly1*^−/−^ mice. The decrease rates of oxytocin peptides in *Ngly1*^−/−^ mice were 78% (male) and 66% (female) compared to those of *Ngly1*^+/+^ mice (Fig. 4a). Down-regulation of oxytocin peptides was also detected in the pituitary gland of *Ngly1*^−/−^ mice, which is the secretory tissue of oxytocin. The decrease rates were 79% (male) and 76% (female) compared to those of *Ngly1*^+/+^ mice (Fig. 4b). At that timing, down-regulation of oxytocin peptides was not detected in the plasma of *Ngly1*^−/−^ mice, probably due to the diurnal variation and rapid clearance of plasma oxytocin (Supplementary Fig. 1). Therefore, plasma oxytocin levels were repeatedly measured every 27 h four times in 7-week-old male and female *Ngly1*^−/−^ mice. Although statistical significance was not detected at most intervals, plasma oxytocin levels showed a tendency to be decreased in *Ngly1*^−/−^ mice compared to *Ngly1*^+/+^ mice (Fig. 4c). Down-regulation of oxytocin peptides was further confirmed in the hypothalamus and pituitary gland of 29-week-old male *Ngly1*^−/−^ mice to increase the credibility of oxytocin down-regulation (Supplementary Fig. 2). In all experiments, oxytocin levels were not significantly changed in *Ngly1*^+/−^ mice compared to *Ngly1*^+/+^ mice.

### Oxytocin levels in *Ngly1*^−/−^ mice after intranasal administration of oxytocin

**Fig. 5:**
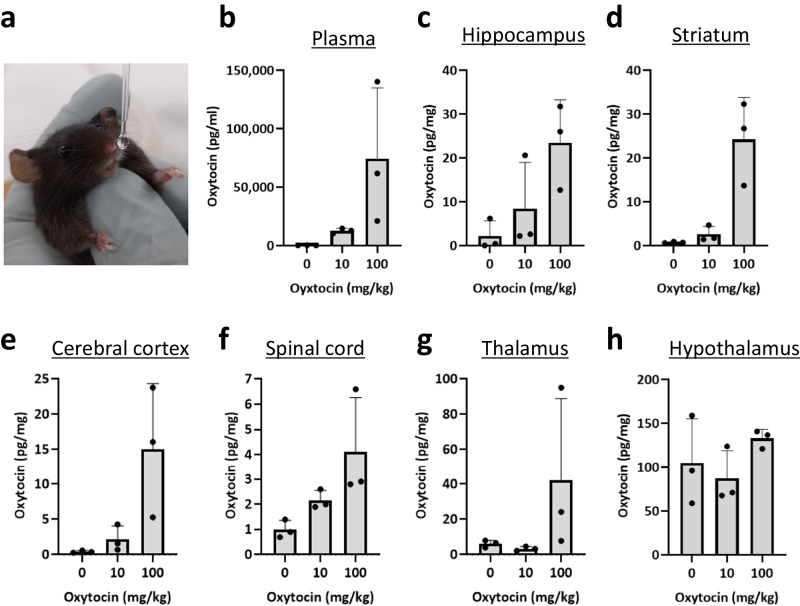
Oxytocin levels in the plasma, brain, and spinal cord of *Ngly1*^−/−^ mice after intranasal administration of oxytocin. Oxytocin (10, 100 mg/kg) or saline was intranasally administered to female *Ngly1*^−/−^ mice (17 or 18-week-old) under anesthesia with 2.5% isoflurane (**a**). **b** Plasma, **c** hippocampus, **d** striatum, **e** cerebral cortex, **f** spinal cord, **g** thalamus, and **h** hypothalamus were collected 5 min after the single administration. Oxytocin levels were measured using enzyme immunoassay. Data represent mean + standard deviation and individual values (*N* = 3).

Delivery of intranasal oxytocin to the central nervous system was confirmed using 17 to 18-week-old female *Ngly1*^−/−^ mice. Dosing was initiated at 10 or 100 mg/kg (6000 or 60,000 IU/kg) due to a lack of effect on seizure-like behaviors in *Ngly1*^−/−^ mice at 1 and 3 mg/kg in our preliminary study. Five minutes after the single intranasal administration, oxytocin levels were increased in the plasma, hippocampus, striatum, cerebral cortex, and spinal cord of *Ngly1*^−/−^ mice, suggesting that this regimen could be applied when evaluating the effects of oxytocin on seizure-like behaviors in *Ngly1*^−/−^ mice (Fig. 5a–f). An increasing trend was also observed in the thalamus, whereas it was not observed in the hypothalamus, probably because the endogenous oxytocin levels were relatively high compared to that in other tissues (Fig. 5g, h).

### Transient suppression of seizure-like behaviors by intranasal oxytocin in *Ngly1*^−/−^ mice

**Fig. 6:**
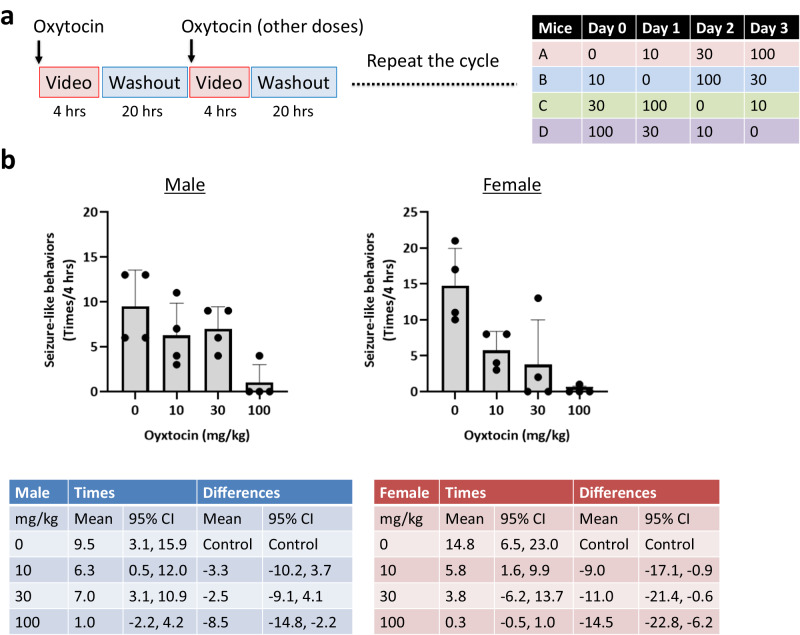
Transient suppression of seizure-like behaviors by intranasal oxytocin administration *in Ngly1*^*−/−*^ mice. **a** Experimental schedule of crossover studies. **b** Oxytocin (0, 10, 30, 100 mg/kg in saline) was intranasally administered to 12-week-old male or female *Ngly1*^−/−^ mice under anesthesia with 2.5% isoflurane. Seizure-like behaviors were monitored for 4 h. This assessment was carried out with a crossover design with washout periods of 20 h. Data represent mean + standard deviation and individual values (*N* = 4). The estimated differences in seizure-like behaviors between oxytocin-treated groups (10, 30, 100 mg/kg) and control group (0 mg/kg) and their adjusted two-sided 95% confidence intervals (CI) were calculated.

Effects of intranasal oxytocin administration on seizure-like behaviors in *Ngly1*^−/−^ mice were evaluated in crossover studies using 12-week-old male and female *Ngly1*^−/−^ mice (Fig. 6a). Seizure-like behaviors in male and female *Ngly1*^−/−^ mice were transiently suppressed by intranasal oxytocin administration. The differences of mean episodes per 4 h-light phase (and two-sided 95% confidence interval) compared to control group (0 mg/kg) were −3.3 (−10.2, 3.7) at 10 mg/kg, −2.5 (−9.1, 4.1) at 30 mg/kg, −8.5 (−14.8, −2.2) at 100 mg/kg in male *Ngly1*^−/−^ mice and −9.0 (−17.1, −0.9) at 10 mg/kg, −11.0 (−21.4, −0.6) at 30 mg/kg, −14.5 (−22.8, −6.2) at 100 mg/kg in female *Ngly1*^−/−^ mice, respectively (Fig. 6b).

## Discussion

Although epileptic seizure is one of the most severe symptoms in patients with NGLY1 deficiency, preclinical studies have not been carried out due to the lack of animal models for epileptic seizures in NGLY1 deficiency. Here we firstly clarified that *Ngly1*^−/−^ mice exhibited several seizure-like behaviors spontaneously (Fig. 1, Supplementary Movie 1). This finding enabled us to analyze the disease mechanism of epileptic seizure in NGLY1 deficiency and explore treatment options.

Significant decreases in oxytocin transcripts were detected in the thalami of male *Ngly1*^−/−^ mice (Fig. 2). Oxytocin is a hypothalamic neuropeptide known for its role in parturition^18,19^, lactation^20^, and social interaction^21^. Oxytocin is also reported to play an important role in the maintenance of brain homeostasis by regulating stress-related hormones, such as corticotropin-releasing hormone, adrenocorticotropic hormone and corticosterone, in the hypothalamic–pituitary–adrenal (HPA) axis^22–25^. Dysregulation of these hormones in HPA axis is a risk factor for epileptic seizure^26^. Therefore, it was speculated that the downregulation of oxytocin in *Ngly1*^−/−^ mice could cause the dysregulation of HPA axis and led to seizure-like behaviors (Fig. 1). The facts that seizure-like behaviors were not detected in *Ngly1*^+/−^ mice (Fig. 1) and that oxytocin was not significantly decreased in *Ngly1*^+/−^ mice (Figs. 3 and 4) also reinforced our hypothesis.

To evaluate the effect of oxytocin on seizure-like behaviors in *Ngly1*^−/−^ mice, oxytocin must be delivered to the central nervous system (CNS) of *Ngly1*^−/−^ mice. Delivery of oxytocin to the CNS had already been demonstrated by intranasal administration in mice^27^, rats^28^, rhesus macaques^29^, and patients with autism spectrum disorder^30^. Therefore, we selected intranasal administration of oxytocin and confirmed its delivery to the CNS in *Ngly1*^−/−^ mice (Fig. 5).

Seizure-like behaviors in *Ngly1*^−/−^ mice were transiently suppressed by single intranasal administration of oxytocin (Fig. 6). The safety of single or chronic intranasal oxytocin administration has been investigated in several clinical studies. For example, no side-effect was detected in school-age children (8–12 years old) 90 min and 24 h after a single administration^31^; chronic administration (twice daily for four weeks) was well-tolerated in generally healthy older men (55–95 years old)^32^. Based on these clinical studies, oxytocin has the potential to be administered to patients with NGLY1 deficiency by carefully optimizing the dosing regimen.

This study has some limitations. While the down-regulation of oxytocin in the hypothalamus of *Ngly1*^−/−^ mice was clarified by bulk RNA-seq analyses, the detailed mechanism of oxytocin down-regulation by *Ngly1* deletion could not be clarified by our subsequent single-nucleus RNA-seq analyses in the hypothalamus of *Ngly1*^−/−^ mice. Other approaches are required to clarify this mechanism.

In conclusion, we observed spontaneous seizure-like behaviors in *Ngly1*^−/−^ mice and the down-regulation of oxytocin in the hypothalamus of *Ngly1*^−/−^ mice. Our results also demonstrated the suppressive effects of intranasal oxytocin administration on seizure-like behaviors in *Ngly1*^−/−^ mice. These findings suggest the therapeutic potential of oxytocin for epileptic seizures in patients with NGLY1 deficiency and would contribute to the clarification of the disease mechanism.

## Methods

### Approval of animal experiments

All animal care procedures and experiments conformed to the Association for Assessment and Accreditation of Laboratory Animal Care guidelines and were approved by the Institutional Animal Care and Use Committee of Takeda Pharmaceutical Co., Ltd. (Kanagawa, Japan).

### Establishment and breeding of *Ngly1*^−/−^ mice

Homozygous *Ngly1* knockout (*Ngly1*^−/−^) mice, heterozygous *Ngly1* knockout (*Ngly1*^+/−^) mice, and wild-type (*Ngly1*^+/+^) littermates were established previously by mating C57BL/6 background *Ngly1*^+/−^ mice with Japanese fancy mouse 1 background *Ngly1*^+/−^ mice to rescue the embryonic lethality of *Ngly1*^−/−^ mice^15^. These mice were bred and provided by Axcelead Drug Discovery Partners (Kanagawa, Japan).

### Video analysis of seizure-like behaviors in *Ngly1*^−/−^ mice

Mice were habituated to single housing for one day in clear acrylic cages (W15 cm, D15 cm, H25 cm) with vents and water insertion slots. The next day, videos were recorded for 24 h from the top of the cage using an infrared camera. Seizure-like behaviors were manually counted using HDWriterAE5.4 software (Panasonic, Osaka, Japan) in a blinded manner by Orizuru Therapeutics (Kanagawa, Japan). All types of seizure-like behaviors were counted as one regardless of the degree and duration. These video analyses were repeated using the same mice at 4 and 10 weeks old.

### Transcriptome analyses in the brain tissues and spinal cord of *Ngly1*^−/−^ mice

The cerebellum, hippocampus, thalamus, striatum, cerebral cortex, and spinal cord were collected from 4- and 10-week-old male *Ngly1*^+/+^ and *Ngly1*^−/−^ mice (*N* = 3). Total RNA was extracted from each tissue using an RNeasy Mini QIAcube kit (QIAGEN, Hilden, Germany). RNA sequencing was performed on the DNBSEQ platform in Azenta. Transcriptome data was analyzed by Axcelead Drug Discovery Partners. Differentially expressed genes were identified using the edgeR (3.1.1) and limma (3.46.0) R packages. In brief, gene expression counts were initially transformed to counts per million (CPM). Subsequently, genes with low expression, where the minimum log-CPM was less than or equal to zero, were removed. Data were then subjected to the trimmed mean of M-values normalization.

### Quantification of oxytocin and other transcripts using real-time PCR

Total RNAs were extracted from the hypothalamus of 10-week-old male and female *Ngly1*^+/+^, *Ngly1*^+/−^ and *Ngly1*^−/−^ mice using the RNeasy Mini QIAcube kit (*N* = 7, combined two experiments). The RNAs were reverse-transcribed using the High-Capacity RNA to cDNA kit (ThermoFisher Scientific, Tokyo, Japan). Transcripts of oxytocin, vasopressin, CRH, and MAP2 were quantified using a 7900HT Fast Real-Time PCR system (ThermoFisher Scientific). Primers, probes, and standard oligos for quantitative PCR were designed using Primer Express^TM^ software v3.0.1 (ThermoFisher Scientific), and their sequences are listed in Supplementary Tables 2 and 3.

### Measurement of oxytocin peptides using enzyme immunoassay

Plasma was collected every 27 h four times from the left ventricles of 7-week-old male and female *Ngly1*^+/+^, *Ngly1*^+/−^, and *Ngly1*^−/−^ mice (*N* = 3) under anesthesia with 2.5% isoflurane. The hypothalamus and pituitary gland were extracted after euthanasia from 10-week-old male and female *Ngly1*^+/+^, *Ngly1*^+/−^, and *Ngly1*^−/−^ mice (*N* = 5). Oxytocin levels were measured by a chemiluminescent enzyme immunoassay in ASKA Pharma Medical Co., Ltd. (Kanagawa, Japan).

### Intranasal administration of oxytocin

Oxytocin peptide (CAS number 50-56-6) was purchased from AA Blocks (San Diego, CA, USA) and dissolved in saline at a maximum concentration of 200 mg/mL. Female *Ngly1*^−/−^ mice were anesthetized with 2.5% isoflurane and intranasally administered with oxytocin using a manual pipette at a volume of 0.5 ml/kg. Oxytocin solutions were prepared in Protein LoBind Tubes (Eppendorf, Hamburg, Germany) just before the intranasal administration.

### Crossover studies to evaluate the effects of oxytocin on seizure-like behaviors

Oxytocin (0, 10, 30, 100 mg/kg in saline) was intranasally administered to 12-week-old male and female *Ngly1*^−/−^ mice. Seizure-like behaviors were counted just after the single administration for 4 h, as previously described. This assessment was carried out with a crossover design with washout periods of 20 h.

### Statistical analyses

Unless otherwise specified, the results were analyzed using EXSUS2014 software version 8.0, SAS 9.3 TS Levle1M2 (SAS Institute, Inc., NC). Limma package, with its voom method^33^, was used to identify differentially expressed genes. Thresholds for identification were a *p* value less than 0.05 and an absolute log-FC (fold change) greater than 1. Bayes algorithm was used to test the statistical significance of differences between *Ngly1*^+/+^ and *Ngly1*^−/−^ mice. The estimated differences in seizure-like behaviors between oxytocin-treated groups (10, 30, 100 mg/kg) and the control group (0 mg/kg) and their adjusted two-sided 95% confidence intervals were calculated to evaluate effects of oxytocin on seizure-like behaviors in male and female *Ngly1*^−/−^ mice. The sample size was chosen based on our preliminary studies with similar methods.

### Reporting summary

Further information on research design is available in the Nature Portfolio Reporting Summary linked to this article.

### Supplementary information

Supplementary Information

Description of Additional Supplementary Materials

Supplementary Movie 1

Supplementary Data 1

Reporting Summary

## Data Availability

All data supporting the findings described in this manuscript are available in the Supplementary Data and from the corresponding author upon reasonable request. *Ngly1*^−/−^ mice, *Ngly1*^+/−^ mice, and *Ngly1*^+/+^ littermates will be available upon reasonable request under MTA.

## Code availability

Transcriptome data were analyzed using a publicly available algorithm. https://www.bioconductor.org/packages/devel/workflows/vignettes/RNAseq123/inst/doc/limmaWorkflow.html.
