# Supplementary material for: Intranasal oxytocin suppresses seizure-like behaviors in a mouse model of NGLY1 deficiency
Source: Commun Biol. 2024 Apr 22;7:460. doi: 10.1038/s42003-024-06131-7 (PMC11035592; doi:10.1038/s42003-024-06131-7)
Supplement: Supplementary file 1 — Supplementary Information [file 42003_2024_6131_MOESM1_ESM.pdf]

Supplementary Information

Supplementary Table 1 | Excerpted list of the Gene Ontology enrichment analysis in 4 and 10-week-old male *Ngly1*$^{-/-}$ mice

Term
chemical synaptic transmission (GO:0007268)
chemical synaptic transmission, postsynaptic (GO:0099565)
excitatory chemical synaptic transmission (GO:0098976)
inhibitory chemical synaptic transmission (GO:0098977)
neuron projection (GO:0043005)
neuron-neuron synaptic transmission (GO:0007270)
phenylethylamine metabolic process involved in synaptic transmission (GO:0090507)
regulation of hormone secretion (GO:0046883)
regulation of N-methyl-D-aspartate selective glutamate receptor activity (GO:2000310)
response to corticosteroid (GO:0031960)
response to glucocorticoid (GO:0051384)
serotonin binding (GO:0051378)
serotonin receptor activity (GO:0004993)
serotonin receptor activity (GO:0099589)
serotonin receptor signaling pathway (GO:0007210)
spontaneous synaptic transmission (GO:0098814)
synaptic transmission (GO:0007268)
synaptic transmission, cholinergic (GO:0007271)
synaptic transmission, dopaminergic (GO:0001963)
synaptic transmission, noradrenergic (GO:0099155)
synaptic transmission, serotonergic (GO:0099153)

Supplementary Table 2 | List of standard oligos for quantitative PCR

	Standard oligo (5' to 3')
Oxytocin	CTGCCTGCTTGGCTTACTGGCTCTGACCTCGGCCTGCTACATCCAGAACTGCCCCCT GGGCGGCAAGAGGGCTGTGCTGGACCTGGATATGCGCAAGTGTCTCCCCTGC
Vasopressin	CACTACGCTCTCCGCTTGTTTCCTGAGCCTGCTGGCCTTCTCCTCCGCCTGCTACTT CCAGAACTGCCCAAGAGGCGGC
CRH	CCCAGCAACCTCAGCCGGTTCTGATCCGCATGGGTGAAGAATACTTCCTCCGCCT GGGGAATCT
MAP2	CGCACTCCTCCAAAGTCCCCAGCTACTCCTAAGCAGCTTCGGCTTATTAACCAA CCACTGCCGGACCTG

Supplementary Table 3 | List of primers and probes for quantitative PCR

	Forward primer (5' to 3')	FAM Probe (5' to 3')	Reverse primer (5' to 3')
Oxytocin	TGCTTGGCTTACTGGCTCTGA	CATCCAGAACTGCCC	GGAGACACTTGCGCATATCCA
Vasopressin	CGCTCTCCGCTTGTTTCCT	CCTGCTGGCCTTC	CTCTTGGGCAGTTCTGGAAGTAG
CRH	CAACCTCAGCCGGTTCTGA	CCGCATGGGTGAAGA	CCCCAGGCGGAGGAAGTA
MAP2	TCCTCCAAAGTCCCCAGCTA	TCCTAAGCAGCTTCGG	CCGGCAGTGGTTGGTTAATAA

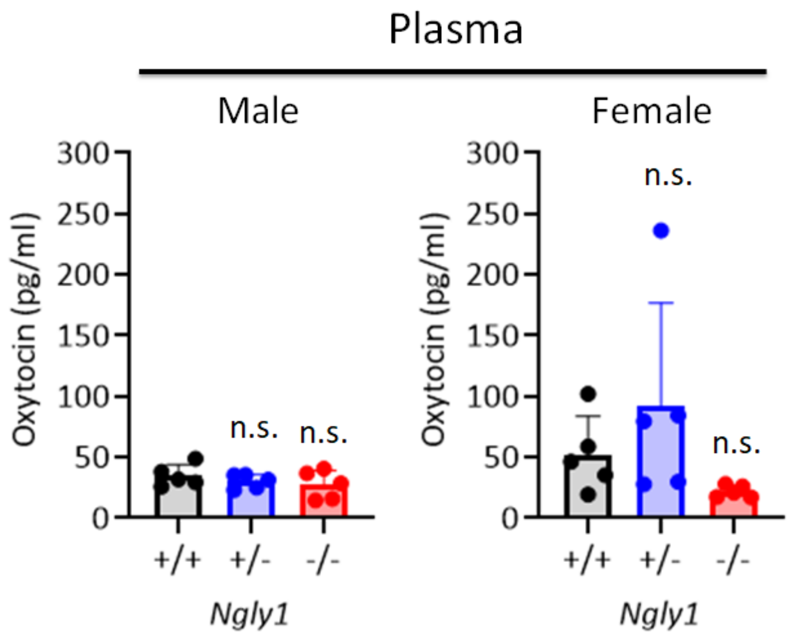

Supplementary Figure 1| Oxytocin levels in the plasma of 10-week-old male *Ngly1*$^{+/+}$, *Ngly1*$^{+/-}$, and *Ngly1*$^{-/-}$ mice. Plasma was collected from 10-week-old male or female *Ngly1*$^{+/+}$, *Ngly1*$^{+/-}$, and *Ngly1*$^{-/-}$ mice. Oxytocin levels were measured by enzyme immunoassay. Data represents mean + standard deviation and individual values (N=5). n.s., not significant vs. *Ngly1*$^{+/+}$ by Dunnett's test.

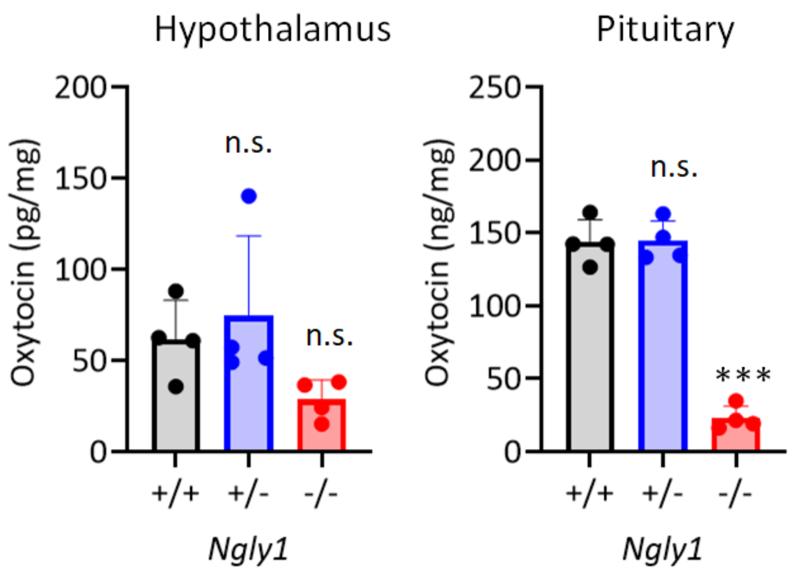

Supplementary Figure 2| Oxytocin levels in the hypothalamus and pituitary gland of 29-week-old male *Ngly1*$^{+/+}$, *Ngly1*$^{+/-}$, and *Ngly1*$^{-/-}$ mice. Hypothalamus and pituitary gland were collected from 29-week-old male *Ngly1*$^{+/+}$, *Ngly1*$^{+/-}$, and *Ngly1*$^{-/-}$ mice. Oxytocin levels were measured by enzyme immunoassay. Data represents mean + standard deviation and individual values (N=4). n.s., not significant; ***, $p<0.001$ vs. *Ngly1*$^{+/+}$ by Dunnett's test.
