# Supplementary material for: Intranasal oxytocin suppresses seizure-like behaviors in a mouse model of NGLY1 deficiency
Source: Commun Biol. 2024 Apr 22;7:460. doi: 10.1038/s42003-024-06131-7 (PMC11035592; doi:10.1038/s42003-024-06131-7)
Supplement: Supplementary file 2 — Description of Additional Supplementary Materials [file 42003_2024_6131_MOESM2_ESM.docx]

**Description of Additional Supplementary Files**

**File name:** Supplementary Data 1

**Description:** The numerical source data for graphs and charts

**File name:** Supplementary Movie 1

**Description:** This movie shows seizure-like behaviors in Ngly1- /- mice.
