# Supplementary material for: Intranasal oxytocin suppresses seizure-like behaviors in a mouse model of NGLY1 deficiency
Source: Commun Biol. 2024 Apr 22;7:460. doi: 10.1038/s42003-024-06131-7 (PMC11035592; doi:10.1038/s42003-024-06131-7)
Supplement: Supplementary file 5 — Reporting Summary [file 42003_2024_6131_MOESM5_ESM.pdf]

nature portfolio

Corresponding author(s): Tadashi Suzuki

Last updated by author(s): Mar 28, 2024

Reporting Summary

Nature Portfolio wishes to improve the reproducibility of the work that we publish. This form provides structure for consistency and transparency in reporting. For further information on Nature Portfolio policies, see our Editorial Policies and the Editorial Policy Checklist.

Statistics

For all statistical analyses, confirm that the following items are present in the figure legend, table legend, main text, or Methods section.

n/a	Confirmed	
☐	☒	The exact sample size (*n*) for each experimental group/condition, given as a discrete number and unit of measurement
☐	☒	A statement on whether measurements were taken from distinct samples or whether the same sample was measured repeatedly
☐	☒	The statistical test(s) used AND whether they are one- or two-sided<br>*Only common tests should be described solely by name; describe more complex techniques in the Methods section.*
☒	☐	A description of all covariates tested
☒	☐	A description of any assumptions or corrections, such as tests of normality and adjustment for multiple comparisons
☐	☒	A full description of the statistical parameters including central tendency (e.g. means) or other basic estimates (e.g. regression coefficient) AND variation (e.g. standard deviation) or associated estimates of uncertainty (e.g. confidence intervals)
☐	☒	For null hypothesis testing, the test statistic (e.g. *F*, *t*, *r*) with confidence intervals, effect sizes, degrees of freedom and *P* value noted<br>*Give P values as exact values whenever suitable.*
☒	☐	For Bayesian analysis, information on the choice of priors and Markov chain Monte Carlo settings
☒	☐	For hierarchical and complex designs, identification of the appropriate level for tests and full reporting of outcomes
☒	☐	Estimates of effect sizes (e.g. Cohen's *d*, Pearson's *r*), indicating how they were calculated

Our web collection on statistics for biologists contains articles on many of the points above.

Software and code

Policy information about availability of computer code

Data collection	Primers, probes, and standard oligos for qPCR were designed using Primer ExpressTM software v3.0.1 (ThermoFisher Scientific),
Data analysis	Seizure-like behaviors were manually counted using HDWriterAE5.4 software (Panasonic, Osaka, Japan)<br>the results were analyzed using EXSUS2014 software version 8.0, SAS 9.3 TS Levle1M2 (SAS Institute, Inc., NC)

For manuscripts utilizing custom algorithms or software that are central to the research but not yet described in published literature, software must be made available to editors and reviewers. We strongly encourage code deposition in a community repository (e.g. GitHub). See the Nature Portfolio guidelines for submitting code & software for further information.

Data

Policy information about availability of data

All manuscripts must include a data availability statement. This statement should provide the following information, where applicable:

- Accession codes, unique identifiers, or web links for publicly available datasets
- A description of any restrictions on data availability
- For clinical datasets or third party data, please ensure that the statement adheres to our policy

All data supporting the findings described in this manuscript are available in the Supplementary Data and from the corresponding author upon reasonable request. Ngly1−/− mice, Ngly1+/− mice, and Ngly1+/+ littermates will be available upon reasonable request under MTA.

Human research participants

Policy information about studies involving human research participants and Sex and Gender in Research.

Reporting on sex and gender	This information has not been collected.
Population characteristics	*Describe the covariate-relevant population characteristics of the human research participants (e.g. age, genotypic information, past and current diagnosis and treatment categories). If you filled out the behavioural & social sciences study design questions and have nothing to add here, write "See above."*
Recruitment	*Describe how participants were recruited. Outline any potential self-selection bias or other biases that may be present and how these are likely to impact results.*
Ethics oversight	*Identify the organization(s) that approved the study protocol.*

Note that full information on the approval of the study protocol must also be provided in the manuscript.

Field-specific reporting

Please select the one below that is the best fit for your research. If you are not sure, read the appropriate sections before making your selection.

☒ Life sciences ☐ Behavioural & social sciences ☐ Ecological, evolutionary & environmental sciences

For a reference copy of the document with all sections, see nature.com/documents/nr-reporting-summary-flat.pdf

Life sciences study design

All studies must disclose on these points even when the disclosure is negative.

Sample size	The sample size was chosen based on our preliminary studies with similar methods.
Data exclusions	No data were excluded from the analyses.
Replication	All attempts at replication were successful.
Randomization	Mice were allocated into experimental groups by their body weight.
Blinding	Seizure-like behaviors were counted in a blinded manner by an outsource vendor.

Reporting for specific materials, systems and methods

We require information from authors about some types of materials, experimental systems and methods used in many studies. Here, indicate whether each material, system or method listed is relevant to your study. If you are not sure if a list item applies to your research, read the appropriate section before selecting a response.

Materials & experimental systems

n/a	Involved in the study	
☒	☐	Antibodies
☒	☐	Eukaryotic cell lines
☒	☐	Palaeontology and archaeology
☐	☒	Animals and other organisms
☒	☐	Clinical data
☒	☐	Dual use research of concern

Methods

n/a	Involved in the study	
☒	☐	ChIP-seq
☒	☐	Flow cytometry
☒	☐	MRI-based neuroimaging

Animals and other research organisms

Policy information about studies involving animals; ARRIVE guidelines recommended for reporting animal research, and Sex and Gender in Research

Laboratory animals	Homozygous Ngly1 knockout (Ngly1−/−) mice, heterozygous Ngly1 knockout (Ngly1+/−) mice, and wild-type (Ngly1+/+) littermates at

Laboratory animals	4 to 29-week-old.
Wild animals	The study does not involve wild animals.
Reporting on sex	Bulk RNA-seq (Figure 2) was applied only to male. Other data were collected using both male and female.
Field-collected samples	The study does not involve samples collected from the field.
Ethics oversight	All animal care procedures and experiments conformed to the Association for Assessment and Accreditation of Laboratory Animal Care guidelines and were approved by the Institutional Animal Care and Use Committee of Takeda Pharmaceutical Co., Ltd. (Kanagawa, Japan).

Note that full information on the approval of the study protocol must also be provided in the manuscript.
